# A Synthetic Phased Array Surface Acoustic Wave Sensor for Quantifying Bolt Tension

**DOI:** 10.3390/s120912265

**Published:** 2012-09-07

**Authors:** Jairo Martinez, Alper Sisman, Onursal Onen, Dean Velasquez, Rasim Guldiken

**Affiliations:** Department of Mechanical Engineering, University of South Florida, 4202 E Fowler Ave., ENB 118, Tampa, FL 33620, USA; E-Mails: jairo@mail.usf.edu (J.M.); alpersisman@mail.usf.edu (A.S.); onursalonen@mail.usf.edu (O.O.); dvelasqu@mail.usf.edu (D.V.)

**Keywords:** bolt tension, real area of contact, non-destructive testing, surface acoustic wave sensor, synthetic phase array, ultrasonic imaging

## Abstract

In this paper, we report our findings on implementing a synthetic phased array surface acoustic wave sensor to quantify bolt tension. Maintaining proper bolt tension is important in many fields such as for ensuring safe operation of civil infrastructures. Significant advantages of this relatively simple methodology is its capability to assess bolt tension without any contact with the bolt, thus enabling measurement at inaccessible locations, multiple bolt measurement capability at a time, not requiring data collection during the installation and no calibration requirements. We performed detailed experiments on a custom-built flexible bench-top experimental setup consisting of 1018 steel plate of 12.7 mm (½ in) thickness, a 6.4 mm (¼ in) grade 8 bolt and a stainless steel washer with 19 mm (¾ in) of external diameter. Our results indicate that this method is not only capable of clearly distinguishing properly bolted joints from loosened joints but also capable of quantifying how loose the bolt actually is. We also conducted detailed signal-to-noise (SNR) analysis and showed that the SNR value for the entire bolt tension range was sufficient for image reconstruction.

## 1. Introduction

Threaded fasteners are one of the most versatile methods for assembling structural components. They are used in many types of physical assemblies, ranging from small toys to large-scale civil infrastructures. Even in an assembly of a single structure, bolts with wide ranging sizes and material properties are actively used. For example, for bridges, large bolts are used for fixing base columns and small bolts are used for attaching maintenance access ladders. As expected, not all bolts are critical for the safe operation of the structure. Relatively low load-bearing fasteners used in large quantities are not monitored and maintained carefully due to cost and feasibility constraints. However, even “non-critical” bolts can cause significant structural problems, even catastrophic failure, if many of them fail.

Loosening of threaded fasteners is a common cause of structure failure. This phenomenon is commonly referred as “slip” at the thread-plate and head-plate interface [1–7]. Loosened bolts are problematic for the clamping force (CF) of the joint. CF is the parameter that defines the effectiveness of a joint. Insufficient CF may lead to joint separation. In the case of separated bolted joints, aggressive fatigue is induced on the remaining bolts due to increase in the tensional stress [2,5]. In addition, loosened bolts tend to loosen even faster than properly tensioned bolts leading to accelerated bolt loosening as the structure ages [2]. Although CF is a critical parameter, direct measurement of CF is very challenging. Instead, bolt tension is typically used to quantify the clamping force in the joint. CF is proportional to the bolt tension by the combined stiffness of the clamped members and the fasteners. Tension of all the fasteners in a joint builds up the common force that ensures clamping. Hence, bolt tension of all the fasteners present in a joint is critical for CF [2,5].

Currently, there are four common methodologies used to control and quantify bolt tension: torque control, turn-of-nut method, direct preload control and stretch control. Torque control is by far the most frequently used method for bolt tension. In this methodology, manual, pneumatic or hydraulic torque wrenches are used to apply wide range of torque to the bolt [2]. The inherent dependency of this method on various variables such as friction factor, torsion, bending and plastic deformation of the threads reduce the measurement accuracy of the applied tension to as low as 30% [2,8]. Turn-of-nut control method has two stages. In the first stage, the bolt is tightened with a conventional torque wrench until it reaches approximately 75% of the material ultimate strength [2]. The second stage involves an additional turn of 180° after the initial tightening. Every 360° degree that the bolt rotates increases the bolt length (and hence the tension) by the bolt pitch, so the final turn ensures a tension that creates stresses larger than the bolt material yield point [2]. Tension accuracy of 5% is reported with this methodology, however this method can only be used for bolts made out of ductile materials with long and well-defined elastic deformation regions [2]. Direct preload control method uses direct estimators of the tension such as strain, stress or deformation. Judiciously positioned strain gages can measure the strain very precisely leading to tension estimation with an accuracy of as high as 1% [2]. Bolt stretch control is another way to quantify the bolt tension. This method employs the transit time of ultrasound measurements along the bolt length to quantify the bolt tension. The main advantage of this methodology is that measurement is independent of friction between the fastener and the clamped plate. Avoiding the friction permits it to be almost as accurate as the instrument used to measure the bolt length change [2]. Additionally, this approach makes it possible to monitor the fastener tension levels only by comparing the new length of the bolt to the value recorded during installation [2]. However, as expected, many older structures with no recorded bolt lengths during installation cannot be evaluated with this method. Also, length variations due to irregular surfaces, uneven machinated processes, temperature changes, plastic deformations and bending displacements introduce significant error to the length estimation.

The common issues with all these frequently used methodologies are their limitation to quantify the bolt tension only during the assembly, measurement capability for a single bolt at a time, relatively high cost and frequent calibration requirement. However, critical bolts should be monitored real-time and single bolt measurement at a time is a significant limitation for large structures with high number of bolts. Response analysis of induced vibrations may be used to characterize the general state of bolted joints. For instance, statistical manipulation can be employed to obtain changes in the vibration signals due to bolt preload variation [9,10]. Recently, signal processing algorithms based on empirical mode decomposition (EMD) have been used to detect changes in vibration signals produced by impact hammers [11]. This method was also validated empirically and by Finite Element Analysis (FEM). Vibration can be used to monitor common problems such as stability or resistance in a structure, but identifying the location of the problem is a fairly difficult task. Local approaches such as monitoring the tension of individual fasteners can be employed [12–15]. The deformations on the fastener were measured by using automatic digital image correlation (ADIC) [12]. Also, piezoelectric sensors were installed in fasteners to sense changes in the electromechanical impedance of the bolts [15]. Guided ultrasonic waves were also used to measure bolt tension. Modulation in Lamb waves due to loosened bolts was used to calculate a joint damage index [13]. Transformation between the wave modes due to stress was exploited by [14] with the purpose of calculating stress levels, tension and CF in the threaded joints. These methodologies often require a direct contact with each bolt to be inspected. Furthermore, majority of the methods require either disassembly of the components or data collected during the installation in order to establish the tension levels.

In this study, we investigate the use of surface acoustic waves (SAW) to quantify bolt tension without contacting the bolt. The advantages of SAW as compared to other acoustic modes are that they propagate over long distances with little amplitude loss enabling large area inspection with minimal number of sensors reducing the cost and complexity [16]. Secondly, SAWs are capable of propagating through curves surfaces and to inaccessible locations, simplifying the design. Also, SAWs are sensitive to many different kinds of flaws. Here, real contact area (RAC), which grows with increased bolt tension, is quantified by its acoustic signature response to SAWs. RAC formed in-between the bolt head and the clamped elements were imaged using a synthetic phase array. SAWs are generated and sensed by off-the shelf bulk piezoelectric transducers connected to a custom-designed ultrasonic wedge. In this paper, we first describe the methodology developed to quantify bolt tension. We, then, discuss the design of experiments including the experimental setup. Finally, we present and discuss the experimental results obtained with the synthetic phased array surface acoustic wave sensor.

## 2. Methodology

**Figure 1.**
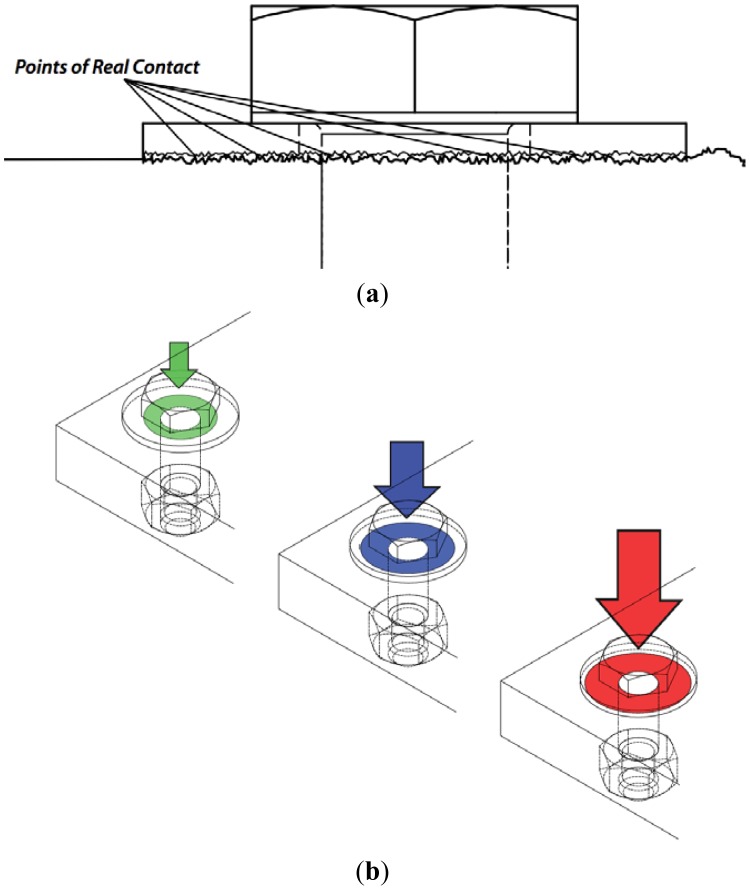
(**a**) Schematic representation of the real contact area of a clamped plate and washer; (**b**) schematic representation of change in the real area of contact as a function of increasing bolt tension.

The interaction of various acoustic wave modes with the boundaries of rough surfaces has been widely studied [17–22]. SAWs interact with solid elements in the surface of the propagation medium [8]. The interaction (for instance with the bolted joint) takes place at the points where the propagation surface and the external body are directly in contact with each other, referred as points of real contact. As illustrated in the Figure 1(a), the points of real contact correspond to the tips of the surface peaks in both materials making direct contact with each other and this represent only a small portion of the entire apparent area of contact. Real area of contact (RAC) is the sum of all these individual contact points [23–25].

It has been reported that the number and size of points of contacts increase with increasing tension in the bolt [24,25]. Figure 1(b) illustrates a schematic representation of the RAC growth due to increase in bolt tension. As can be observed from this figure, the higher the tension (signified with “arrow”), the larger the RAC between the plate and the washer (signified with the “donuts”). The RAC is, in fact, different from the apparent area of contact, but at high-tension levels the difference between those becomes minimized [24]. The saturation of the RAC at a specific preload level establishes the maximum tension level that should be applied to the bolt for optimal and safe operation. The way in which the RAC grows is of special interest for estimating the tension. As illustrated in Figure 1(b), the RAC grows from the center of the washer towards the washer perimeter. This behavior can be explained by analyzing the way the tension is applied to the bolt head. First, the preload transforms into a pulling force exerted to the head center. The surface peaks closer to the center are then affected more than the ones in the perimeter and therefore the expansion of the RAC can be predicted as propagating radially outward.

**Figure 2.**
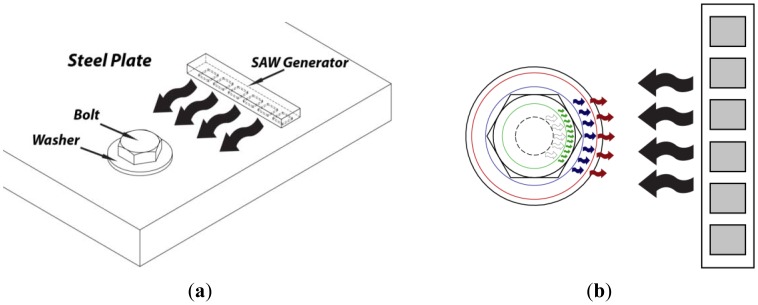
(**a**) Schematic representation of the proposed methodology used to quantify bolt tension; (**b**) representation of surface acoustic wave propagation and reflection from four different boundaries. White arrows represent no load, green arrows low load, blue arrows medium load and red arrows high load. The black arrows represent the incoming acoustic wave.

**Figure 3.**
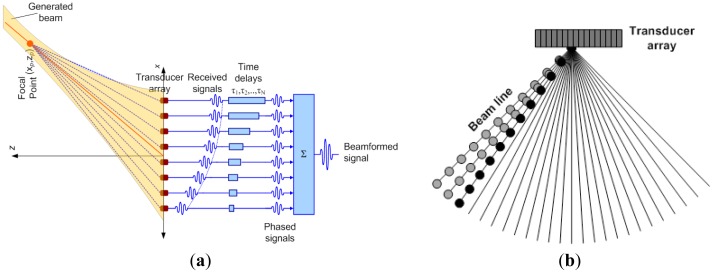
Scanning the interested area using transducer array. (**a**) Construction of a beamformed signal; (**b**) Scanning by constructing successive beamlines.

In this study, the interaction of the SAW with the bolt and RAC was investigated by a set-up illustrated in Figure 2(a). Waves created by a SAW generator are directed towards a bolted joint. As the tension at the bolt is increased, the position of the reflective boundary is expected to move from the edge of the joint towards the outer washer perimeter, as presented in Figure 2(b). It is hypothesized that the increase in the bolt tension results in a proportional increase in the RAC, represented by the circles in Figure 2(b). The arrows represent the incident (black) and reflected (colored) waves. The white, green, blue and red colored arrows represent the SAW reflections from the plate hole and the RAC corresponding to tension states of low, medium and high intensity respectively. The method employs a phased array to obtain the B-scan ultrasonic image of the bolted joint in order to quantify the RAC, which is proportional to tension level in the bolted joint. Ultrasonic image of the bolt assembly is obtained by generating SAW pulses and receiving the echoes using the array elements. Each beam line in the image is constructed by summing the beamformed signals. The construction of image is illustrated in Figure 3 where the τ_1_ to τ_N_ represent the proper delays and the output is the beamformed signal corresponding to focal point. The delay profile for each focal point in the beam line is calculated using the following equation:

(1) $$\tau_{i}=\frac{1}{c}\left[R_{p}-{\left((x_{i}-x_{p})+z_{p}\right)}^{1/2}\right]$$

where *c* is the SAW velocity, (*x_i_*,0) is the coordinate of array element, (*x_p_*,*z_p_*) is the coordinates of the focal point on the beam line and *R_p_* is the distance between the array center and focal point. SAW velocity (∼2,900 m/s) was calculated by the Rayleigh wave equation [26], and verified by ultrasonic delay time (pulse-receive) experiments performed on the 1018 steel plate by using two transducers. The delay that is calculated for each transducer element basically eliminates the differences of flight time that is the travel duration from focal point to the each array element. The sum of properly delayed signals results in an in-phase sum called beamformed signal. The mathematical expression to determine the beamformed signal is given in Equation (2). Here *A_i_* is the aperture function applied to each receive and transmit element, *s_i,j_* is the received signal corresponding to *i*th transmit and *j*th receive element, *τ_i_* is refer to transmit and receive delays [27]. Dynamic focusing is employed in receive mode, hence each beam line is obtained by summing the beamformed signals obtained by focusing to each radial point [28]. The combined beamlines form the image of the interested area (illustrated in Figure 3(b)).

(2) $$B(t)=\sum_{i=1}^{N}A_{tx,i}\sum_{j=1}^{N}A_{rx,j}s_{i,j}(t-\tau_{tx,i}-\tau_{tx,j}+\frac{2R_{p}}{c})$$

The location of bolt boundary is obtained using the ultrasonic image by measuring the distance between the boundary and the array center since it is proportional to the RAC. The distance is an important parameter to determine the health of the bolted joint.

## 3. Experimental Design

**Figure 4.**
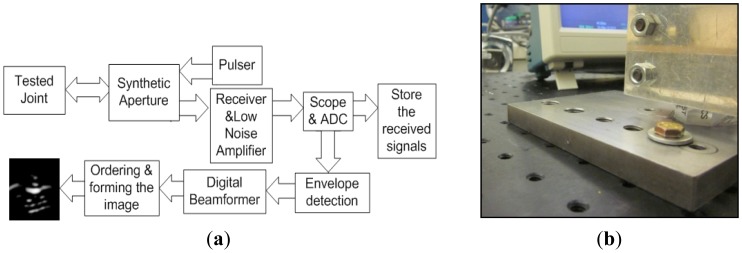
(**a**) The block diagram and (**b**) the photographic illustration of the experimental setup.

The proposed concept was investigated by experimental studies. The experimental setup is illustrated by the block diagram (Figure 4(a)). The tested joint was composed of a 1018 steel plate of 12.7 mm (½ in) thickness, a 6.4 mm (¼ in) grade 8 bolt and stainless steel washer with 19 mm (¾ in) of external diameter, as illustrated in Figure 4(b). The joint was lubricated with general purpose oil in order to facilitate the tightening process. The wideband excitation pulses were generated and the echoes were received and amplified by a pulsar-receiver (Olympus 5072PR, Olympus NDT Inc., Waltham, MA, USA). The received signals were displayed/digitized by a scope (Tektronix TDS 2024B, Textronix Inc., Beaverton, OR, USA). The digitized signals are stored in a computer and the image is generated using a custom-designed MATLAB code.

We obtained the ultrasound images using simpler synthetic array imaging system instead of more involved phased array system for the proof of the proposed concept studies. Synthetic array imaging is chosen due to the simplified beamformer structure although it may degrade the image quality by lowering the signal to noise ratio (SNR).

**Figure 5.**
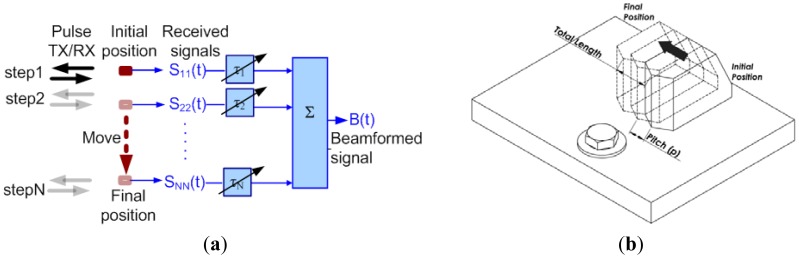
(**a**) Illustration of the synthetic aperture imaging and (**b**) the experimental implementation.

Synthetic phased array was formed by a single angle beam transducer actuated from judicious several positions within a linear array. The wideband SAW pulse is transmitted and the echo is received in each movement step (Figure 5(a)). Consequently N-element linear array is synthetically formed using a single moving transducer. It should be noted that synthetic array imaging cannot achieve all of the TX/RX combinations; the transmitted array element must receive the echo. The missing transmit-receive (TX/RX) combinations resulting in decreased SNR as compared to phased imaging modality. However, the major advantage of synthetic phased array modality is significantly simplified beamformer since the total number of TX/RX combinations is decreased dramatically [27–31]:

(3) $$B(t)=\sum_{i=1}^{N}A_{rx,i}A_{tx,i}s_{i,i}\left(t-2\tau_{i}+\frac{2R_{p}}{c}\right)$$

In this study, we used a 5 MHz bulk piezoelectric transducer (C541-SM, Olympus NDT Inc.) with circular aperture of 12.7 mm (½ in) attached to an ultrasonic wedge that is specifically designed for converting bulk pressure wave (P-wave) into SAWs in steel (ABWML-5T, Olympus NDT Inc.). Ultrasonic couplant (Soundsafe, Sonotech Inc., Glenview, IL, USA) was used at the surfaces between the wedge and the transducer and between the wedge and the test piece to facilitate the transmission of ultrasonic energy. The transducer-wedge system is linearly moved to realize synthetic array. The position control of the angle beam transducer (transducer-wedge attachment) was achieved through a manual translator stage (PT3, Thorlabs Inc., Newton, NJ, USA) consisting of micrometers with resolution of 25 μm and maximum displacement range of 25.4 mm. The transducer and the wedge were adapted to the translator stage by custom designed aluminum adaptors. In Figure 5(b), two important features of the synthetic linear array are illustrated: array pitch (p) and total array length. In this study, the array pitch was selected as 250 μm corresponding to less than half the wavelength. With this selection criterion, sufficient lateral resolution was achieved and formation of side lobes in the acoustic beam was avoided [30]. The number of elements in the array determines the array size which is proportional to the lateral resolution of the system. In this study, an array with 50 elements was used corresponding to the total array length of 12.5 mm that results in 1.25 mm beam diameter at 29 mm away from the center of the array. Note that 29 mm corresponds to the distance between the transducer array and the bolted joint. This 1.25 mm beam diameter provides sufficient resolution for imaging the bolted joint.

We investigated the proposed methodology with several bolt tension values for comparison. During the experiments, the torque applied to the bolt was precisely controlled as it is indirectly related to the bolt tension. Torque and tension are related by the bolt diameter and a friction factor which is a function of screw type and the materials used [5]. However, it is worth noting that for a particular bolt installed in a specific plate, the torque and tension are directly proportional. Also as RAC is directly proportional to tension, torque and RAC values are also proportional to each other:

(4) $$\text{T}\propto {\text{F}}_{\text{T}}\;\text{and RAC}\propto {\text{F}}_{\text{T}}\;\text{then RAC}\propto \text{T}$$

where *T* is the torque applied to the bolt, *F_T_* is the bolt tension. The theoretical value of maximum torque that can be applied to the bolt is given in the Equation (5) below [5,30]:

(5) $$T=0.85KA_{t}S_{y}d$$

where *K* is the friction factor, *A_t_* is the tensile stress area, *S_y_* is the bolt yield strength, *d* is the bolt nominal diameter and *T* is the torque applied. For the 6.3 mm (¼ in) grade 8 bolt used in experiments, the values of the parameters *K* and *S_y_* are 0.18 and 691 N/mm^2^ with the tensile stress area and nominal diameter being 20.5 mm^2^ and 6.3 mm respectively [29]. By substituting these values into Equation (5), the maximum allowable torque for the bolt can be obtained as 13.78 Nm. In this study, five torque values equally spaced from 0 Nm to 13.78 Nm are selected for conducting the experiments. A torque wrench with 29 Nm of maximum capacity and 0.11 Nm of resolution was used to apply the required torque.

## 4. Results and Discussion

**Figure 6.**
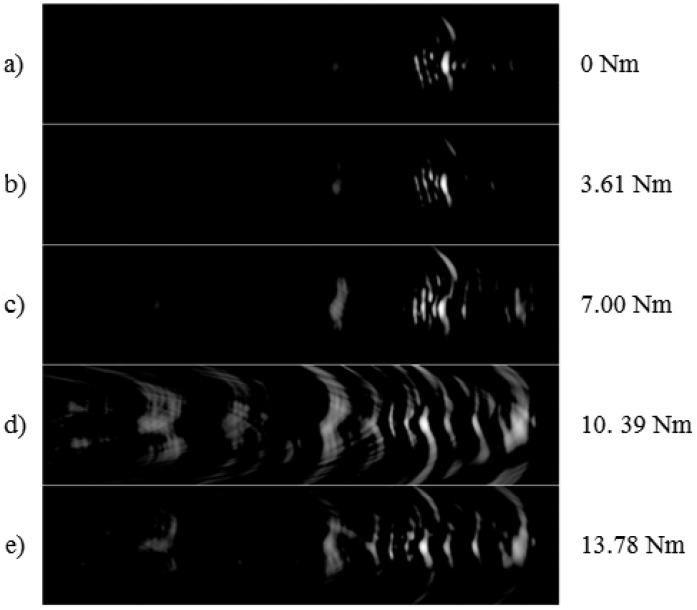
Images reconstructed with dynamic range of 15 dB with the SAW reflections from a 6.3 mm (¼ in) bolt with a stainless steel washer. Reconstructed ultrasonic images for five different torque values are illustrated.

The results were obtained for five different torque levels (with 3.61 Nm torque increments) using the experimental set-up detailed in the previous section. Figure 6 illustrates the experimentally obtained reconstructed images with these different torque levels. In this figure, the horizontal (range) and the vertical extensions of each image are 2.37 × 2.54 cm, respectively. The range extension of the image was 0.92 cm to 3.29 cm and the vertical extension was from −1.27 cm to +1.27 cm where the transducer array is located at the origin. As can be observed from this figure, there was a clear change in the shape of reconstructed images, especially the location of the first reflection from the bolted joint when different torque values are applied. It was observed that increasing the applied torque resulted in displacement of the first reflection boundary towards the transducer array. Furthermore, even in the reference condition of no torque, a clear reflection from the plate hole was observed. It should also be pointed out that even with a very loose tightening of the bolt (corresponding to torque value 3.61 Nm in Figure 6), a noticeable movement of the reflective boundary towards the left side of the image is observed. As the applied torque is increased, images became noisier due to increase in the real area of contact however there was a similar trend of movement of reflective boundary towards the array. One can also observe that the brightness of the images is increasing as the torque is decreased since the response is getting stronger.

**Figure 7.**
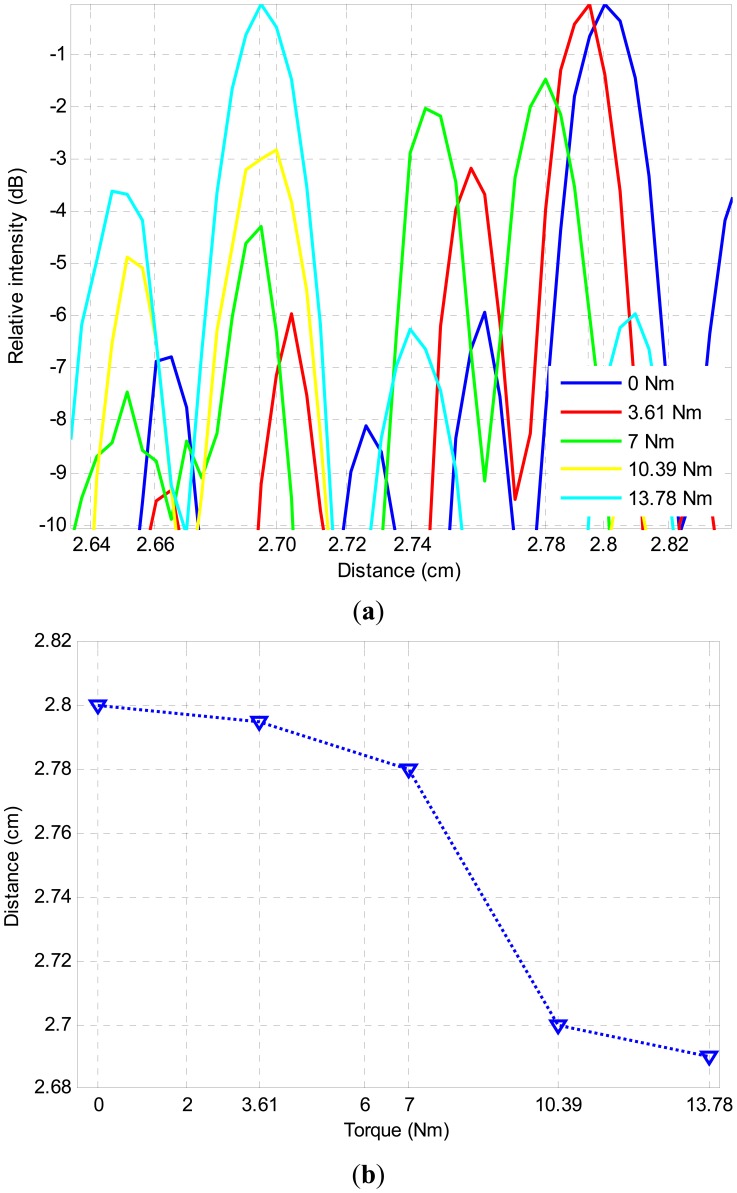
(**a**) Averaged 1-D images of the first reflection boundary with a dynamic range of 6 dB; (**b**) Experimentally obtained position of the first reflection boundary as a function of the torque applied.

To quantify the displacement of the first reflection boundary from the joint center towards the transducer array as a function of applied torque, the relative average signal intensity of the reconstructed images as a function of distance from the transducer array is plotted in Figure 7(a). The 1-D average plot, which is a simplification of the 2-D reconstructed image (Figure 6), is obtained by taking the average of lateral dimension of the 2-D image. The 1-D plot allows establishing the exact position of the maximum intensity point of the 2-D image. The position of this point is used to estimate the actual distance change of the reflective boundary. Figure 7(b) illustrates the position of the first reflection boundary as a function of the applied torque. Firstly, one can clearly observe from the maximum intensities of the reconstructed images that there is a clear trend of moving the reflection boundary towards the transducer array as the torque is increased. It can also be observed from Figure 7(b) that the distance between the position of first reflection boundary with no torque applied and maximum allowable torque of 13.78 Nm is 0.1 cm. The first reflection boundary corresponding to rest of the torque values are positioned between these two extreme values. Note that the first reflection boundary position change does not have a linear trend with increasing torque. This expected behavior can be explained by the fact that with this phased array image reconstruction technique, we are quantifying the location of the first reflection boundary, not the real area of contact (RAC). As a result, the boundary distance and the torque should not be expected to have a linear relationship. It should be pointed out that this non-linear variation of the movement of the first reflection of boundary as a function of applied torque can easily be used to estimate the torque (hence the tightening of the bolt) by just obtaining the distance of the first reflection boundary from the transducer array. Another important characteristic of the Figure 7(b) is the fact that RAC saturation takes place around 10 Nm corresponding to first reflection boundary position of 2.7 cm.

### 4.1. Signal to Noise Ratio

**Figure 8.**
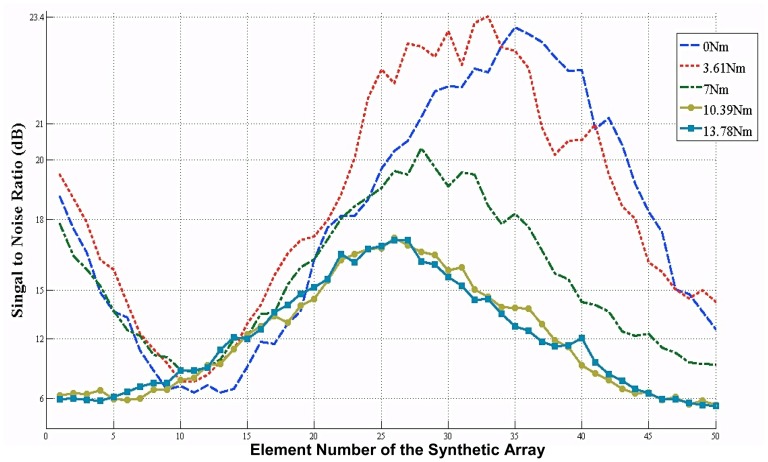
Signal to noise ratio of the bolt reflections based on original signals.

Synthetic phased array modality was used in this study as opposed to phased array modality for significantly simplified beamformer design. However, one drawback of synthetic phased array modality is its lower signal to noise ratio (SNR). In order to ensure that SNR associated with the generated images is sufficient, we conducted a detailed study measuring the SNR of all the received signals (A-scans). In Figure 8, the SNRs of the signals reflected by the bolt are presented. This figure illustrates the SNR levels for the 50 signals corresponding to each 50 positions of the transducer. As expected, higher SNR values were obtained closer to the center position due to the reflections from the washer (or the hole). It was also observed that average SNR decreases with the increasing torque level. As expected, lower SNR values resulted in lower quality images for higher torque levels compared to lower torque values. Lower image quality may potentially lead to less accurate estimation and higher errors.

The minimum dynamic range that can be applied to the images was determined by the averaged SNR. The reflective boundary position was calculated using the 1-D averaged plot based on the location of the signal peaks. Consequently, the peak SNR value of Figure 8 at each torque level corresponds to the peaks in Figure 7(a). The minimum peak SNR of the system was around 17 dB at 13.78 Nm, as shown in Figure 8. The averaged signal to noise ratio for each torque value is depicted in Table 1 for reference. The decrease of SNR can clearly be seen as the applied torque is increased. The average SNR value of the system is around 15 dB. This SNR level was good enough to differentiate the signal from the ambient noise. The reduction of the SNR of the system around the bolt may be attributed to some attenuation due to the bolt interference. This analysis allows identification of sources of errors for further reducing the SNR of the system.

## 5. Conclusions

This study presents the first step towards the development of a bolt tension sensor based on surface acoustic waves. The bolt tension was estimated using the reflection of SAWs created by the bolted joint interference. Our results indicate that this method is not only capable of clearly distinguishing properly bolted joints from loosened joints but also capable of quantifying how loose the bolt is. Increase in the bolt tension resulted in an increase in the number of points of that are in-contact at the bolt-plate interface (real area of contact). This increase in the real area of contact caused the first reflection boundary position change measured from the bolted joint by using surface acoustic waves. The boundary location change was quantified by using a 1-D and 2-D ultrasonic images obtained by linear synthetic array image processing technique.

The 2-D ultrasound imaging allowed clear and detailed visualization of the position of the reflective boundary when applied torque to the bolt was changed. For the entire range of torque values from zero to maximum allowable, as hypothesized, increase in the torque value increased the real area of contact and therefore moved the position of the reflective boundary towards the ultrasonic array. The boundary position *versus* the torque applied to the bolt was also investigated. There were two distinct regions; the first region presented a non-linear behavior, while the second one was identified as a saturation region. The saturation region (presented at the highest torque values) was characterized by negligible change in the boundary position due to further increase in the torque applied. The signal-to-noise (SNR) analysis was also performed and the results indicate that the intensity of the signal was reduced as the torque was increased. However, it should be noted that the SNR value even for the maximum allowable torque value was sufficient for image reconstruction. The bolt tension sensor investigated here has the potential to be used as a component in a non-destructive structural health monitoring (SHM) system for local monitoring of civil infrastructures.

The proposed method has a significant potential to be used in real-life applications. However there maybe limitations that needs to be addressed. Current method utilizes an off-the-shelf wedge (8 cm × 3 cm × 5 cm in size) coupled to surface with an acoustic couplant. Although this fairly small size is suitable for majority of applications, for those applications where the structure does not allow direct access, sensor arrays can be directly integrated to structures. Wireless interrogation can then be employed for data acquisition. Surface condition is also an important factor that may affect the operation. Surface conditions at the plate-bolt and plate-wedge interface (such as contamination, surface roughness) and along the propagation path (such as liquid loading) may possibly cause minor problems such as wave attenuation, undesired scattering. However, it should be noted that these issues can be overcome by using proper commercially available surface treatments and ultrasonic couplants.

## Figures and Tables

**Table 1.** Experimentally obtained signal to noise ratio (SNR) for different torque values applied to the bolted joint.

**Torque Level (Nm)**	0	3.61	7	10.39	13.78
**Average SNR (dB)**	18	18.31	15.75	12	12.05

## References

[1] Hess D.P., Bickford J. (1988). Vibration and shock-induced loosening. Handbook of Bolts and Bolted Joint.

[2] Bickford J.H. (2008). Introduction to the Design and Behavior of Bolted Joints.

[3] Goodier J.N. (1945). Loosening by vibration of threaded fastenings. Mech. Eng..

[4] Sauer J.A., Lemmon D.C., Lynn E.K. (1950). Bolts: How to prevent their loosening's. Mach. Des..

[5] Budynas R.G., Nisbett K. (2006). Screw, fasteners, and the design of nonpermanent joints. Shigley's Mechanical Engineering Design.

[6] Pai N.G., Hess D.P. (2002). Experimental study of loosening of threaded fasteners due to dynamic shear loads. J. Sound Vibrat..

[7] Pai N.G., Hess D.P. (2003). Influence of fastener placement on vibration-induced loosening. J. Sound Vibrat..

[8] Pedinoff M.E., Waldner M., Jones W.R. (1971). Refraction and reflection of surface acoustic waves at boundaries of layered anisotropic substrates: Gold on lithium niobate. J. Appl. Phys..

[9] Huang Y.H., Liu L., Yeung T.W., Hung Y.Y. (2009). Real-time monitoring of clamping force of a bolted joint by use of automatic digital image correlation. Opt. Laser Tech..

[10] Tanner N.A., Wait J.R., Farrar C.R., Sohn H. (2003). Structural health monitoring using modular wireless sensors. J. Intell. Mater. Syst. Struct..

[11] Milanese A., Marzocca P., Nichols J.M., Seaver M., Trickey S.T. (2008). Modeling and detection of joint loosening using output-only broad-band vibration data. Struct. Health Monit..

[12] Esmaeel R.A., Briand J., Taheri F. (2011). Computational simulation and experimental verification of a new vibration-based structural health monitoring approach using piezoelectric sensors. Struct. Health Monit..

[13] Amerini F., Meo M. (2011). Structural health monitoring of bolted joints using linear and nonlinear acoustic/ultrasound methods. Struct. Health Monit..

[14] Kim N., Hong M. (2009). Measurement of axial stress using mode-converted ultrasound. NDT E Int..

[15] Mascarenas D.L., Park G., Farinholt K.M., Todd M.D., Farrar C.R. (2009). A low-power wireless sensing device for remote inspection of bolted joints. J. Aerosp. Eng..

[16] Ballantine D.S. (1997). Acoustic Wave Sensors: Theory, Design, and Physico-Chemical Applications.

[17] Kendall K., Tabor D. (1971). An ultrasonic study of the area of contact between stationary and sliding surfaces. Proc. Roy. Soc. Lond. A Math. Phys. Eng. Sci..

[18] Pau M. (2003). Estimation of real contact area in a wheel-rail system by means of ultrasonic waves. Tribol. Int..

[19] Królikowski J., Szczepek J. (1991). Prediction of contact parameters using ultrasonic method. Wear.

[20] Drinkwater B.W., Dwyer-Joyce R.S., Cawley P. (1996). A study of the interaction between ultrasound and a partially contacting solid-solid interface. Proc. Roy. Soc. Lond. A Math. Phys. Eng. Sci..

[21] Królikowski J., Szczepek J. (1992). Phase shift of the reflection coefficient of ultrasonic waves in the study of the contact interface. Wear.

[22] Takeuchi A. (2004). Establishment of lubrication diagnostic technique with ultrasonic method. J. Jpn. Soc. Tribol..

[23] Johnson K.L. (1985). Contact Mechanics.

[24] Czichos H. (1978). Tribology: A Systems Approach to the Science and Technology of Friction, Lubrication and Wear.

[25] Fischer-Cripps A.C. (2000). Surfaces Forces, Adhesion and Friction.

[26] Auld B.A. (1990). Acoustic Fields and Waves in Solids.

[27] Thomenius K.E. Evolution of Ultrasound Beamformers.

[28] Karaman M., Atalar A., Koymen H. (1992). Optimization of dynamic receive focusing in ultrasound imaging. Acoust. Imag..

[29] Collins J.A., Staab G.H., Busby H.R. (2002). Mechanical Design of Machine Elements and Machines.

[30] Szabo T.L. (2004). Diagnostic Ultrasound Imaging: Inside Out..

[31] Hoskins P.R., Martin K., Thrush A. (2010). Diagnostic Ultrasound: Physics and Equipment.

